# 

**Published:** 1890-12

**Authors:** 


### CONTENTS.

### COMMUNICATIONS.

Cure of Cleft Palate........................................ 577
Do we, in our practice, do the best in our power for our patients?.. 582
Address........................................ 589
Inti nate Dia gn sis of L ssons affecting the Teeth........................................ 598
Report of D scussion on Ohio Sta e Dental Society........................................ 606

### SELECTIONS.

The V lue of the Examin tion of the Te th in Anthropological Research........................................ 609

Female Physicians in E rlier Times........................................ 616

### EDITORIAL.

Glossary of Terms Applied in Electrical Science........................................ 617
What a Dentist has done........................................ 619
Honorable Mention........................................ 619
Ohio State Meeting Jottings........................................ 620
Bibliographical........................................ 621
The Tariff on Dental Goods........................................ 622

